# Case of Ascites

**Published:** 1843-10-14

**Authors:** Robert M. Denig

**Affiliations:** M’Connellsburg, Bedford Co., Pa.


### A CASE OF ASCITES.

BY ROBERT M. DENIG, M. D.

To the Editor of the Medical Examiner.

Dear Sir:—A case of Ascites came under my notice a few weeks since, which from its novelty and unusual extent, I deem not unworthy of reporting to you. The patient was a married female, aged 19, who has never borne any children, and has been labouring under dropsical effusion into the abdomen for three years; was tapped about nine months ago, since which time (the paracentesis) anasarca of the lower extremities and walls of the abdomen has been added to the original disease. On the 17th ult. I was requested, in connection with Drs. Duffield and Stewart, to see the patient. Her appearance was unique in the extreme, and such as precludes all possibility of a description—measuring in circumference over the umbilicus six feet three inches, and the lower end of the sternum being situated in her, relatively, where the nipple is in a well proportioned individual; her breathing attended with the utmost difficulty, and her sufferings indicating beyond doubt that her existence could not under those circumstances be prolonged any considerable length of time; we consequently immediately proceeded to the operation of Paracentesis Abdomines. A strong bandage was adjusted round her, held firmly by assistants, and a trochar of moderate size introduced about three inches below the umbilicus. In consequence of the great thickness of the walls of the abdomen, owing to the anasarca, it was deemed prudent to introduce the trochar to a more than usual depth, and upon the stem being withdrawn, a thick transparent albuminous looking fluid followed, so tenacious that it ran with great difficulty through the canula; and in a few minutes ceased altogether A probe was introduced to free the tube, which answered the purpose to a limited extent, but before half a gallon had been evacuated it again ceased to run. The probe was again passed in, but to no purpose; and, under the impression that the operation would prove abortive from the smallness of the tube and the tenacity of the fluid, it was, upon consultation, resolved to withdraw the instrument and abandon the case as hopeless.

Acting upon this determination I withdrew the canula nearly an inch, when a thin aqueous fluid shot through it, *pleno rivo*, and continued to run without any interruption, for three hours and thirty-five minutes—until the enormous quantity of eighteen gallons were evacuated. After this quantity had been discharged the patient became so faint and feeble that it was found necessary to desist, notwithstanding a large quantity of water, to all appearances, yet remained. The singularity of the entire change in the evacuation, from that of the albuminous, resembling, in appearance and consistency the white of eggs, to that of the aqueous resembling a common simple decoction of coffee—apart from the enormous quantity discharged—renders the case a somewhat novel one, and not devoid of interest. Could there have existed, conjointly, ovarium and abdominal dropsy, and the sac of the former affusion have been perforated by the introduction of the trochar to such an extent, and that being freed from it in a subsequent stage of the operation, it allowed the abdominal effusion alone to escape? Is there any marked difference between the effusion in ascites and ovarium dropsy? Could two fluids so entirely different have existed separately in the same sac? The patient's health, since the operation, has been moderate, and she is enabled to attend her usual avocations without inconvenience. Are there any cases on record in which a much larger quantity of fluid was evacuated?

*M'Connellsburg, Bedford Co., Pa.*, Oct. 3, 1843.

